# New Insight into the Substrate Selectivity of Bovine Milk γ-glutamyl Transferase via Structural and Molecular Dynamics Predictions

**DOI:** 10.3390/molecules28124657

**Published:** 2023-06-09

**Authors:** Lichuang Cao, Cameron J. Hunt, Anne S. Meyer, René Lametsch

**Affiliations:** 1 Department of Food Science, Faculty of Science, University of Copenhagen, 1958 Frederiksberg C, Denmark; lichuang.cao@food.ku.dk; 2 Department of Biotechnology and Biomedicine, Technical University of Denmark, 2800 Kgs. Lyngby, Denmark; cjahu@dtu.dk (C.J.H.); asme@dtu.dk (A.S.M.)

**Keywords:** γ-glutamyl donor, γ-glutamyl acceptor, molecular docking, γ-glutamyl-enzyme intermediate, molecular dynamic simulation

## Abstract

Bovine milk γ-glutamyltransferase (BoGGT) can produce γ-glutamyl peptides using L-glutamine as a donor substrate, and the transpeptidase activity is highly dependent on both γ-glutamyl donors and acceptors. To explore the molecular mechanism behind the donor and acceptor substrate preferences for BoGGT, molecular docking and molecular dynamic simulations were performed with L-glutamine and L-γ-glutamyl-*p*-nitroanilide (γ-G*p*NA) as donors. Ser450 is a crucial residue for the interactions between BoGGT and donors. BoGGT forms more hydrogen bonds with L-glutamine than γ-G*p*NA, promoting the binding affinity between BoGGT and L-glutamine. Gly379, Ile399, and Asn400 are crucial residues for the interactions between the BoGGT intermediate and acceptors. The BoGGT intermediate forms more hydrogen bonds with Val-Gly than L-methionine and L-leucine, which can promote the transfer of the γ-glutamyl group from the intermediate to Val-Gly. This study reveals the critical residues responsible for the interactions of donors and acceptors with the BoGGT and provides a new understanding of the substrate selectivity and catalytic mechanism of GGT.

## 1. Introduction

Because of its ability to catalyze the transpeptidase reaction to produce γ-glutamyl peptides, γ-glutamyl transferase from bovine milk (BoGGT) has recently received increasing attention [1,2,3]. γ-Glutamyl peptides can be applied as taste enhancers to enhance the mouthfeel and aftertaste of food [4,5]. Furthermore, it has been demonstrated that γ-glutamyl peptides exhibit a variety of biological activities, comprising anti-inflammatory, hypoglycemic, and appetite-suppressing effects [6]. In 2009, γ-glutamyl peptides were identified from Swiss Gruyere and Gouda cheeses [7,8]. Toelstede and Hofmann [7] found an association between the transpeptidase activity and concentration of γ-glutamyl peptides in various cheese types. They demonstrated that BoGGT might produce γ-glutamyl peptides in raw milk cheeses. It has been established that γ-glutamyl peptides in Parmesan cheese are mainly produced by the transpeptidase reaction of BoGGT [3]. Moreover, Yang et al. [9] identified γ-glutamyl peptides from protein hydrolysates of enzyme-modified butter and revealed that BoGGT could produce γ-glutamyl peptides. Our previous study identified BoGGT (G3N2D8) using LC-MS/MS and validated its ability to generate γ-glutamyl peptides from whey protein and casein hydrolysates with L-glutamine as a donor [1,2].

The types of γ-glutamyl donors and acceptors affect the transpeptidase activity of GGT. A recent study documented that *Escherichia coli* GGT has stereoselectivity for γ-glutamyl acceptors, suggesting that the transpeptidase activity of *E. coli* GGT depends on the acceptor substrate [10]. Hillmann, Behr, Ehrmann, Vogel and Hofmann [3] proposed that BoGGT has a higher substrate specificity for L-methionine and L-phenylalanine than L-lysine, L-histidine, L-leucine, L-aspartic acid, L-threonine, and L-glutamic acid. The GGT activity is typically measured using a colorimetric method with γ-glutamyl-*p*-nitroanilide (γ-G*p*NA) as a donor [11,12,13,14]. Our previous study showed that BoGGT has a lower transpeptidase activity with γ-G*p*NA as the γ-glutamyl donor than that of a L-glutamine donor; further, the transpeptidase activity of BoGGT is highly dependent on the acceptor with both L-glutamine and γ-G*p*NA donors [2].

However, no previous study has been conducted into the interaction mechanism between BoGGT and its γ-glutamyl donor, as well as the interaction between the BoGGT intermediate and γ-glutamyl acceptor. It is well-established that human γ-glutamyl transferase (GGT) works by sequential mechanisms to catalyze a bi-substrate reaction [10,15]. Firstly, hydroxyl oxygen of threonine in the active site of GGT will initiate a nucleophilic attack for the amide carbon on the donor, forming a γ-glutamyl enzyme intermediate with the γ-glutamyl moiety. Then the γ-glutamyl moiety will transfer from the intermediate to the γ-glutamyl acceptor. γ-Glutamyl peptides will be produced in case the acceptor is a peptide or an amino acid, whereas L-glutamic acid will be generated in case the acceptor is a water molecule [10,15]. It has been established that the catalytic nucleophile of human GGT is Thr381 [16]. Analogously, Thr380 was confirmed to be the catalytic nucleophile in the active site of BoGGT by multiple sequence alignment [1].

To explore the binding mechanism between BoGGT and substrates, molecular docking and molecular dynamics (MD) simulations have been utilized [17,18]. The computational work requires a three-dimensional (3D) structure of BoGGT; however, the 3D structure of BoGGT is unresolved. Recently, it was proposed that AlphaFold could reliably construct 3D structures from protein sequences [19], which could predict the 3D structure of BoGGT. This study aims to (1) explain the donor and acceptor substrate preferences for BoGGT via modeling of the interaction between key active site residues of BoGGT and different donors and acceptors; (2) provide new insight into the catalytic reaction trajectory by molecular dynamics simulations of the BoGGT substrate binding and the bond distance between the key amino acids and substrate reactant in the active site.

## 2. Results and Discussion

### 2.1. Mechanism behind the Donor Selectivity of BoGGT

#### 2.1.1. Prediction of the 3D Structure of BoGGT

**Figure 1:**
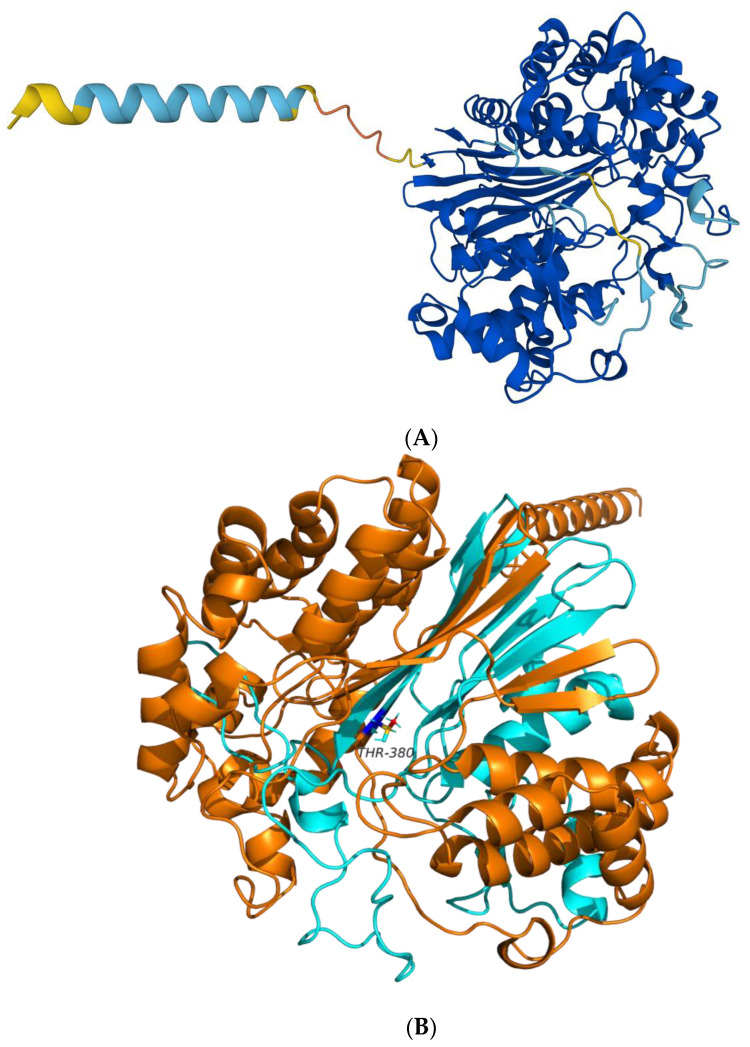
AlphaFold prediction on the three-dimensional structure of bovine milk γ-glutamyl transferase. (**A**) Three-dimensional structure of bovine milk γ-glutamyl transferase displayed in various colors based on model confidence. Note: The per-residue confidence score (pLDDT) is utilized to assign a color to each residue in the model. Residues with extremely high confidence (pLDDT > 90) are shown in blue, while those with high confidence (70 < pLDDT < 90) are shown in cyan. On the contrary, residues with low confidence (50 < pLDDT < 70) are displayed in yellow, while very low confidence residues (pLDDT < 50) are represented in orange. (**B**) Three-dimensional structure of bovine milk γ-glutamyl transferase with light and heavy chains shown in different colors.

Our previous study identified BoGGT as the bovine enzyme (G3N2D8) [1]. In the present study, AlphaFold v2.0 was applied to construct the 3D structure of BoGGT. As illustrated in Figure 1A, the majority of the BoGGT residues were predicted with very high confidence, supporting that the 3D structure was accurately predicted.

#### 2.1.2. Interactions between BoGGT and Donor

The catalytic nucleophile of human GGT has already been reported to be Thr381 [15,16]; multiple sequence alignment confirmed that the catalytic nucleophile of BoGGT is Thr380 [1]. It is generally accepted that the hydroxyl oxygen (-OH) on Thr380 from BoGGT will attack the amide carbon of the donor, resulting in the formation of the γ-glutamyl–BoGGT intermediate [20]. Our previous study demonstrated that BoGGT has lower transpeptidase activity with γ-G*p*NA as a γ-glutamyl donor than the L-glutamine donor [2]. However, the molecular mechanism underlying the difference in donor affinity for BoGGT remains unclear.

**Figure 2:**
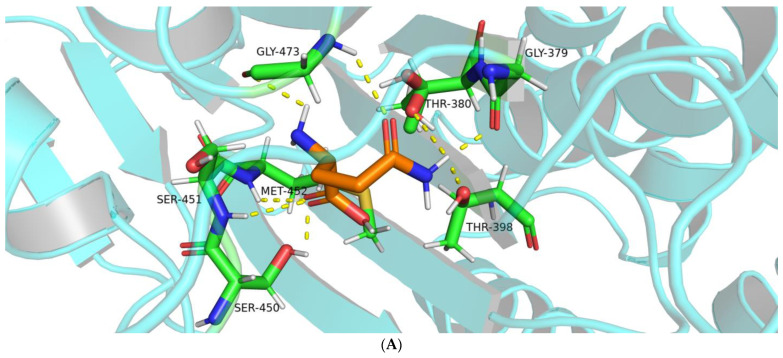

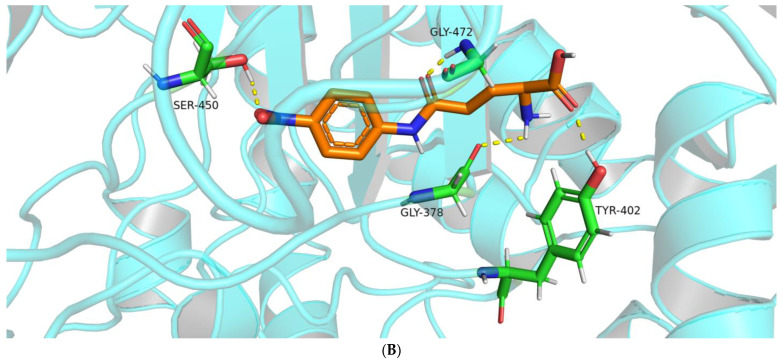
Interactions between γ-glutamyl transferase from bovine milk and γ-glutamyl donor. (**A**) Interactions between bovine γ-glutamyl transferase and L-glutamine. (**B**) Interactions between bovine γ-glutamyl transferase and L-γ-glutamyl-*p*-nitroanilide. Note: The atoms in silver represent hydrogen; the atoms in blue and red represent nitrogen and oxygen, respectively.

Molecular docking results showed eight hydrogen bonds between BoGGT and L-glutamine, whereas there were four hydrogen bonds between BoGGT and γ-G*p*NA. These hydrogen bonds are responsible for glutamine binding at the active site. This might explain why BoGGT has a higher transferase activity with L-glutamine as a γ-glutamyl donor than γ-G*p*NA. Figure 2A displays a PyMol visualization of the interaction between BoGGT and L-glutamine. The oxygen atom on Gly379 and Thr380 formed a hydrogen bond (H-bond) with the L-glutamine’s amide hydrogen atom, respectively. H-bonds were formed between the hydrogen atoms on Ser450, Ser451, and Met452 and the carboxy oxygen atom on L-glutamine. The transpeptidase activity of human GGT was reduced to 1% of the wild-type GGT after site mutagenesis of Ser451 or Ser452 [21], and Ser451 or Ser452 also formed H-bonds with the substrate; this indicates that Ser451 and Ser452 promote the interaction between the active site and substrate. The corresponding residues of Ser451 and Ser452 in human GGT are Ser450 and Ser451 in BoGGT, respectively. Therefore, H-bonds between Ser450, Ser451, and the carboxy oxygen atom on L-glutamine seem to promote the donor substrate binding in the active site, resulting in the formation of the γ-glutamyl–BoGGT intermediate. In addition, the amide hydrogen and carbonyl oxygen atoms on Gly473 formed H-bonds with the L-glutamine’s carbonyl oxygen and amide hydrogen atoms, respectively. It has been proposed that the H-bonds between Gly473 and the donor substrate promote the formation of the γ-glutamyl-GGT intermediate [15].

An H-bond occurs between the oxygen atom on Thr398 and the amide hydrogen on L-glutamine. It is universally accepted that the formation of H-bonds between enzyme and substrates greatly affects the specificity of the enzyme and thus facilitates the enzymatic reaction. PyMol visualization of the interaction between BoGGT and γ-G*p*NA is presented in Figure 2B. The oxygen atom on Gly378 formed an H-bond with the amide hydrogen atom of γ-G*p*NA. The hydrogen atom on Tyr402, Ser450, and Gly472 formed H-bonds with the oxygen atom on γ-G*p*NA. Based on the interactions between BoGGT and the γ-glutamyl donor, it could be concluded that Ser450 is a crucial residue for donor substrate recognition.

#### 2.1.3. MD Simulations of BoGGT–Donor Complex

**Figure 3:**
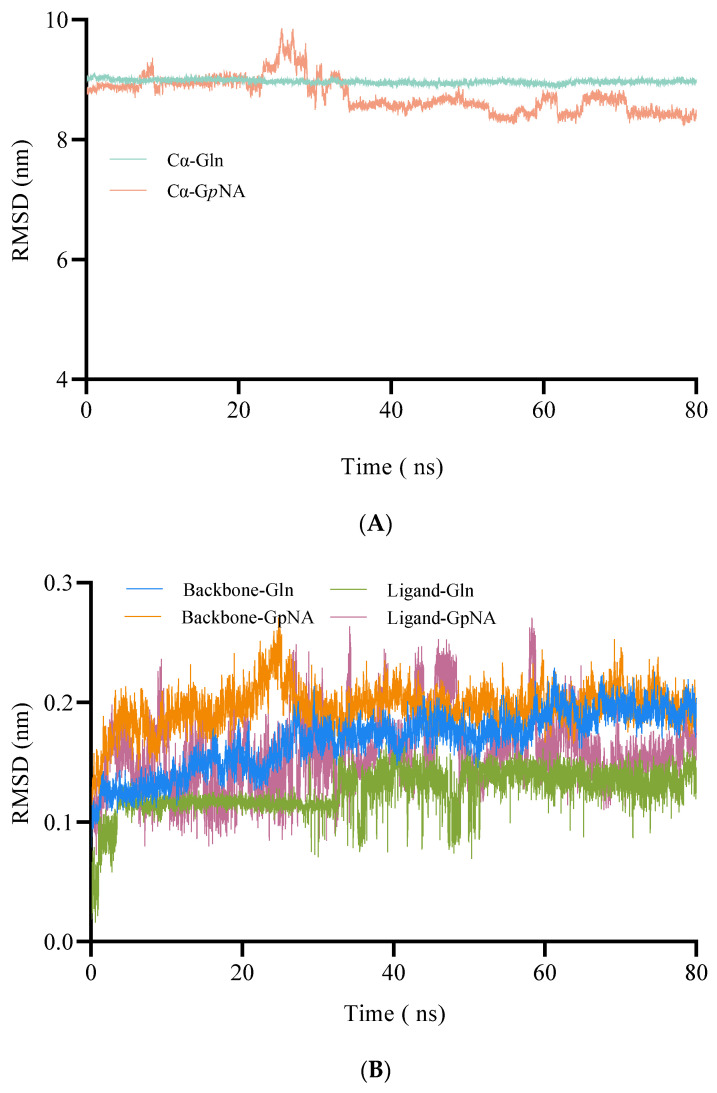

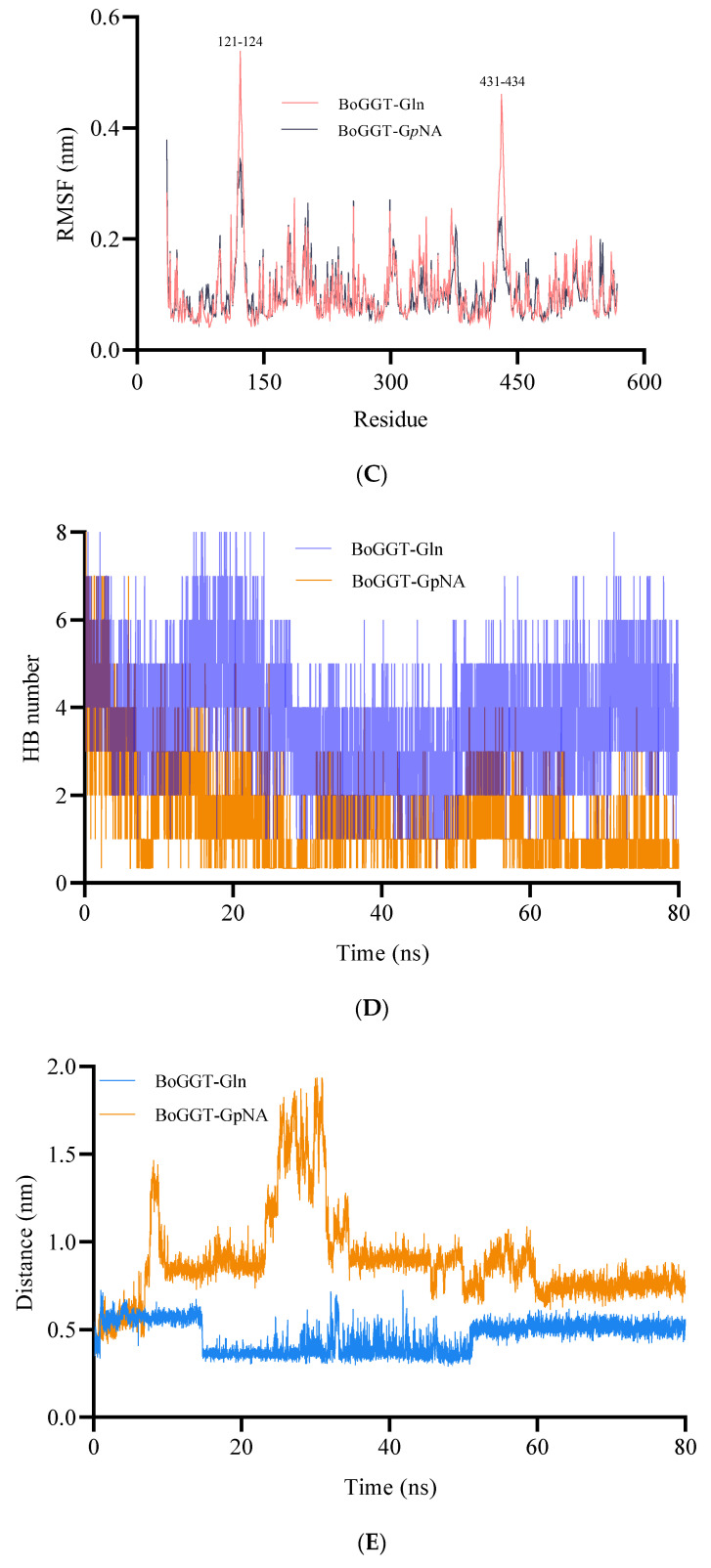
Molecular dynamic simulations of BoGGT–donor complexes. (**A**) Root-mean-square deviation of C_α_ of BoGGT–donor. (**B**) Root-mean-square deviation of BoGGT backbone and donor substrate. (**C**) Root-mean-square fluctuations (RMSFs) of BoGGT from BoGGT–Gln and BoGGT–G*p*NA complexes. (**D**) Hydrogen bond numbers between BoGGT and the donor substrate. (**E**) Distance between the hydroxyl oxygen atom on Thr380 of BoGGT and the amide carbon on the donor substrate.

After the molecular docking, the BoGGT–donor complex was employed to conduct 80 ns MD simulations to explore the interaction between BoGGT and the donor substrate. The root-mean-square deviation (RMSD) represents how structures change over time compared to the starting point. The RMSD of the complexes (C_α_ of BoGGT and the donor substrate) was used to assess the system stability of BoGGT–donor complexes during an 80 ns simulation. As demonstrated in Figure 3A, both complexes had steady RMSDs after 40 ns simulations, indicating that the L-glutamine and the γ-G*p*NA donor were tightly bound to BoGGT. The RMSDs of the donor substrates and backbone of BoGGT were calculated to evaluate the stability of the donor substrate and backbone of BoGGT further (Figure 3B). After a 60 ns simulation, the RMSD of the BoGGT backbone and ligand (L-glutamine and γ-G*p*NA) from complexes stabilized, indicating that the protein had equilibrated. Nevertheless, the RMSDs of the complexes were notably greater than that of the BoGGT backbone, which might be due to the opening and closing motion of a lid from BoGGT observed during the simulations. The above results indicate that the BoGGT–glutamine and BoGGT–G*p*NA complexes reached a steady state after 60 ns of simulations. For each BoGGT–donor substrate complex, the root-mean-square fluctuations (RMSFs) of its constituent amino acids were determined, as well. Amino acids 121–124 and 431–434 of the BoGGT–Glutamine complexes were found to have higher fluctuation values than those of BoGGT–G*p*NA, suggesting these residues might be involved in the conformational alteration of the BoGGT–Glutamine complexes (Figure 3C).

H-bonds between the enzyme and substrate are widely considered to be the primary factor that facilitate the enzymatic reaction [22]. Consequently, H-bond numbers were also utilized to evaluate the binding between the donor substrate and BoGGT. As displayed in Figure 3D, the H-bond numbers between BoGGT and L-glutamine were significantly higher than those between BoGGT and γ-G*p*NA during the 60–80 ns simulation (*p* < 0.05), which was consistent with the docking results. This suggests that BoGGT has a higher interaction with L-glutamine than γ-G*p*NA.

GGT first forms γ-glutamyl–GGT intermediate during the transpeptidase reaction when the hydroxyl oxygen on the active site attacks the carbonyl carbon on the donor substrate [23]. Consequently, during the MD simulations, the distance between the hydroxyl oxygen atom (OG_1_) on Thr380 and the amide carbon (C) on the γ-glutamyl donor was inspected to evaluate the possibility of the interaction between BoGGT and the donor. As displayed in Figure 3E, the distance between OG_1_ on Thr380 and the amide carbon on L-glutamine (0.514 ± 0.031 nm) was significantly (*p* < 0.05) lower than that for BoGGT and γ-G*p*NA (0.749 ± 0.042 nm) during the 60–80 ns simulations.

Previous research has reported that Pro427-Ser438 of human GGT form a lid-loop, which may affect the substrate binding in the active site [24,25,26]. Sequence alignment showed that the lid-loop of BoGGT is Pro426-Ser437, and the opening and closing motion was observed during the MD simulation of BoGGT–donor complexes, which could recognize the donor substrate and regulate the access of the donor to the active site. γ-G*p*NA is a bigger molecule than L-glutamine, allowing L-glutamine to access the active site easily. This could also explain why BoGGT has higher transpeptidase activity with L-glutamine as a donor than γ-G*p*NA. The H-bond numbers between L-glutamine and BoGGT were significantly (*p* < 0.05) greater than those of γ-G*p*NA, which can decrease the OG_1_-C distance and thus promote the binding affinity between BoGGT and L-glutamine.

### 2.2. Mechanism behind the Acceptor Selectivity of BoGGT

#### 2.2.1. Construction and Optimization of the BoGGT Intermediate

**Figure 4:**
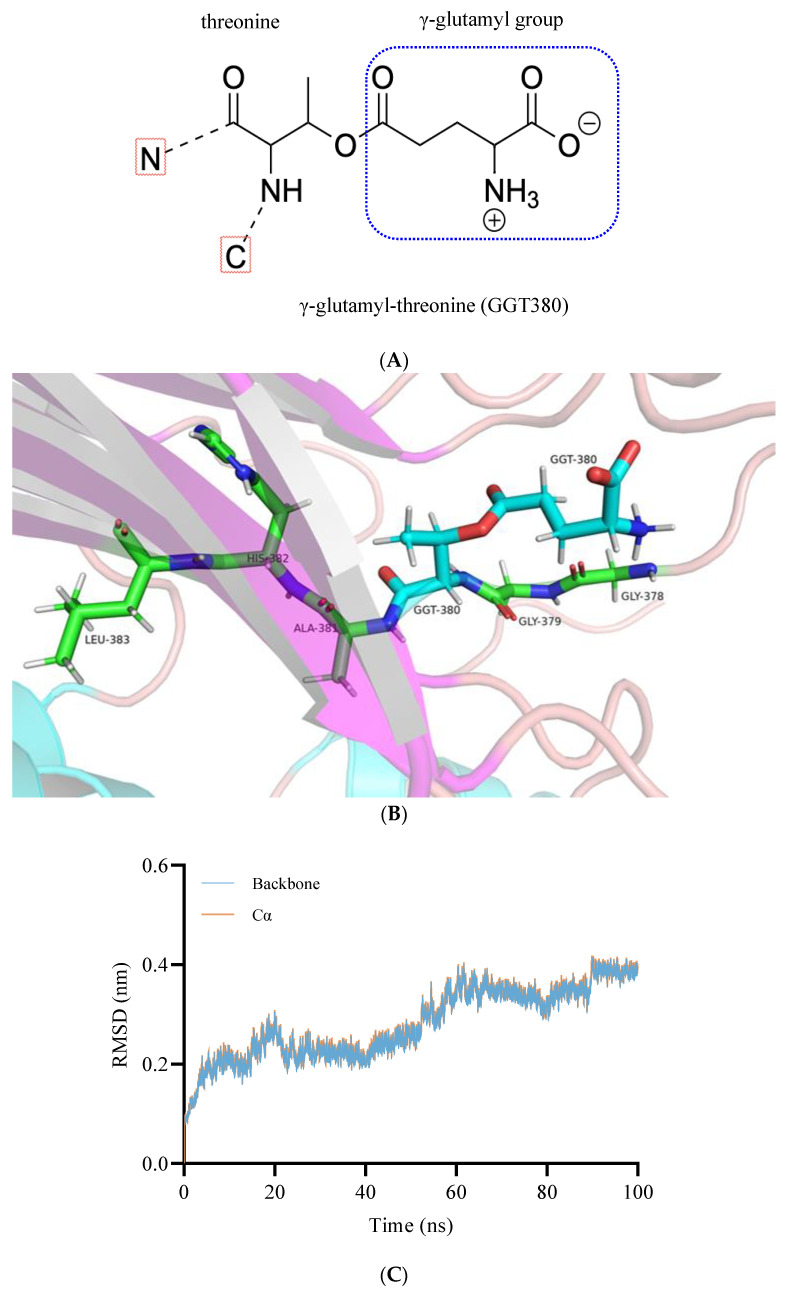
Molecular dynamic simulations of the γ-glutamyl–BoGGT intermediate. (**A**) Diagram of the γ-glutamyl-threonine on the γ-glutamyl–BoGGT intermediate. (**B**) Three-dimensional structure of the γ-glutamyl–BoGGT intermediate. Note: The atoms in silver represent hydrogen; the atoms in blue and red represent nitrogen and oxygen, respectively. (**C**) Root-mean-square deviation of backbone and alpha-carbon from the γ-glutamyl–BoGGT intermediate.

Our previous study revealed that the transpeptidase activity of BoGGT is highly dependent on the acceptor with both L-glutamine and γ-G*p*NA donors [2]. In the second step of the transpeptidation reaction, it is well established that the γ-glutamyl–GGT intermediate reacts with acceptors to produce γ-glutamyl peptides. To explore the interaction mechanism between the γ-glutamyl–BoGGT intermediate and acceptors, the γ-glutamyl–BoGGT intermediate was constructed and optimized using GROMACS. After constructing the γ-glutamyl–BoGGT intermediate, the intermediate was optimized using 100 ns MD simulations. Figure 4B presents the γ-glutamyl–BoGGT intermediate, which is the combination of the hydroxyl oxygen on Thr380 of BoGGT with the γ-glutamyl group from the donor. As demonstrated in Figure 4C, the RMSDs of the intermediate’s backbone and alpha-carbon atom stabilized after 60 ns of simulation, indicating that the 3D structure of BoGGT reached a steady state. The intermediate’s 3D structure at 100 ns was used to perform molecular docking.

#### 2.2.2. Interactions between the BoGGT Intermediate and Acceptors

Our previous study demonstrated that the transpeptidase activity of BoGGT with L-methionine as an acceptor substrate was significantly lower than that of Val-Gly, whereas it was significantly higher than that of L-leucine [2]. Hillmann, Behr, Ehrmann, Vogel and Hofmann [3] also proposed that L-methionine was a better acceptor substrate than L-leucine for BoGGT. However, the molecular mechanism behind the difference in acceptor affinity for the BoGGT intermediate remains unclear. In the current study, molecular docking was utilized to explore the interactions between the BoGGT intermediate and γ-glutamyl acceptors.

**Figure 5:**
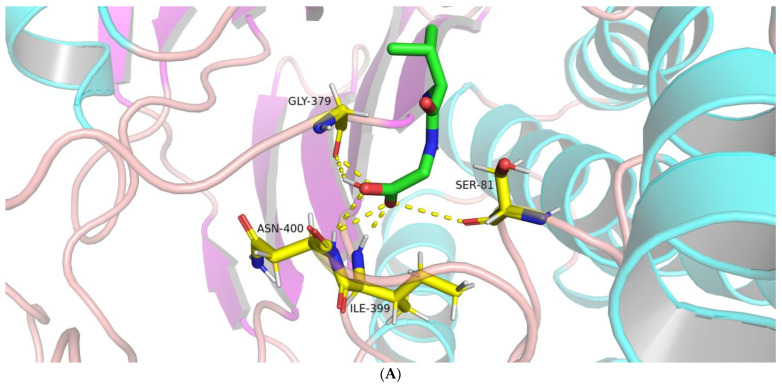

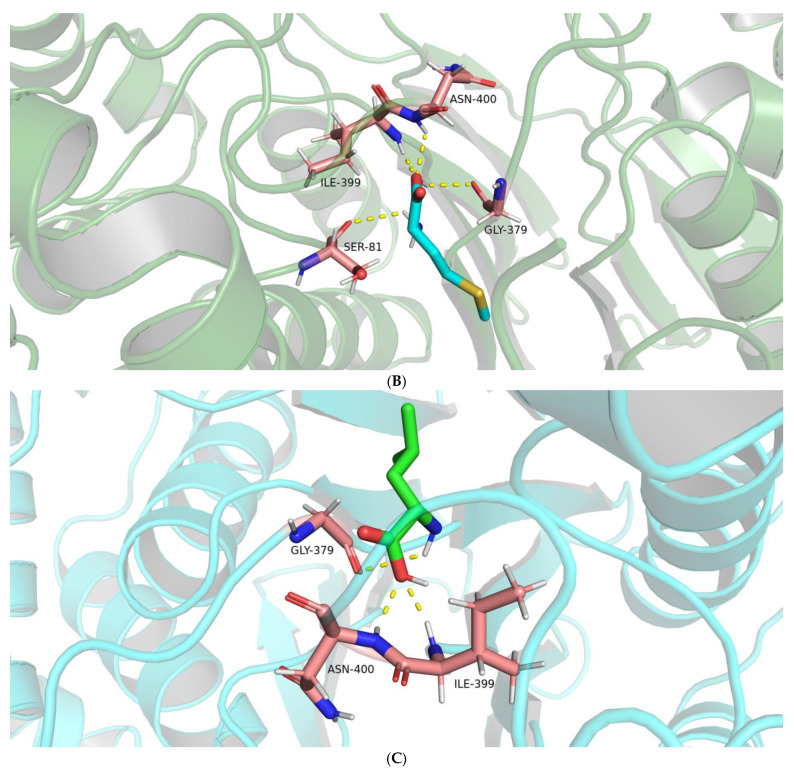
Interactions between the γ-glutamyl–BoGGT intermediate and γ-glutamyl acceptors. (**A**) Interactions between the γ-glutamyl–BoGGT intermediate and Val-Gly. (**B**) Interactions between the γ-glutamyl–BoGGT intermediate and L-methionine. (**C**) Interactions between the γ-glutamyl–BoGGT intermediate and L-leucine. Note: The atoms in silver and yellow represent hydrogen and sulfur, respectively. The atoms in blue and red represent nitrogen and oxygen, respectively.

As shown in Figure 5A, molecular docking of the BoGGT intermediate and Val-Gly showed five hydrogen bonds; three residues (Gly379, Ile399, and Asn400) were involved in the formation of H-bonds. The oxygen atom on Gly379 formed an H-bond with the hydroxyl hydrogen atom on Val-Gly. Two H-bonds were formed between the hydrogen atoms on Ile399 and Asn400, and the carboxyl oxygen atom on Val-Gly, and one H-bond was formed between the hydrogen atom on Asn400 and the hydroxyl oxygen atom on Val-Gly. Four H-bonds were formed between four residues (Ser81, Gly379, Ile399, and Asn400) from the intermediate and L-methionine (Figure 5B), whereas there were three H-bonds between Gly379, Ile399, and Asn400 on the intermediate and L-leucine (Figure 5C).

The hydrogen atom on Ile399 and Asn400 formed one H-bond with the same hydroxyl oxygen atom on L-methionine, respectively. The oxygen atom on Ser81 formed an H-bond with the amide hydrogen atom on L-methionine, and carboxyl oxygen on Gly379 formed an H-bond with the hydroxyl hydrogen atom on L-methionine. Regarding the interaction between the intermediate and L-leucine, the hydrogen atom on Ile399 and Asn400 formed one H-bond with the same hydroxyl oxygen atom on L-leucine, respectively. The carboxyl oxygen on Gly379 formed an H-bond with the amide hydrogen atom on L-leucine. It could be concluded that Gly379, Ile399, and Asn400 are key residues responsible for the interactions between the intermediate and acceptors, thereby regulating the transfer of the γ-glutamyl moiety from the γ-glutamyl–BoGGT intermediate to the acceptor.

#### 2.2.3. MD Simulations of BoGGT Intermediate–Acceptor Complex

**Figure 6:**
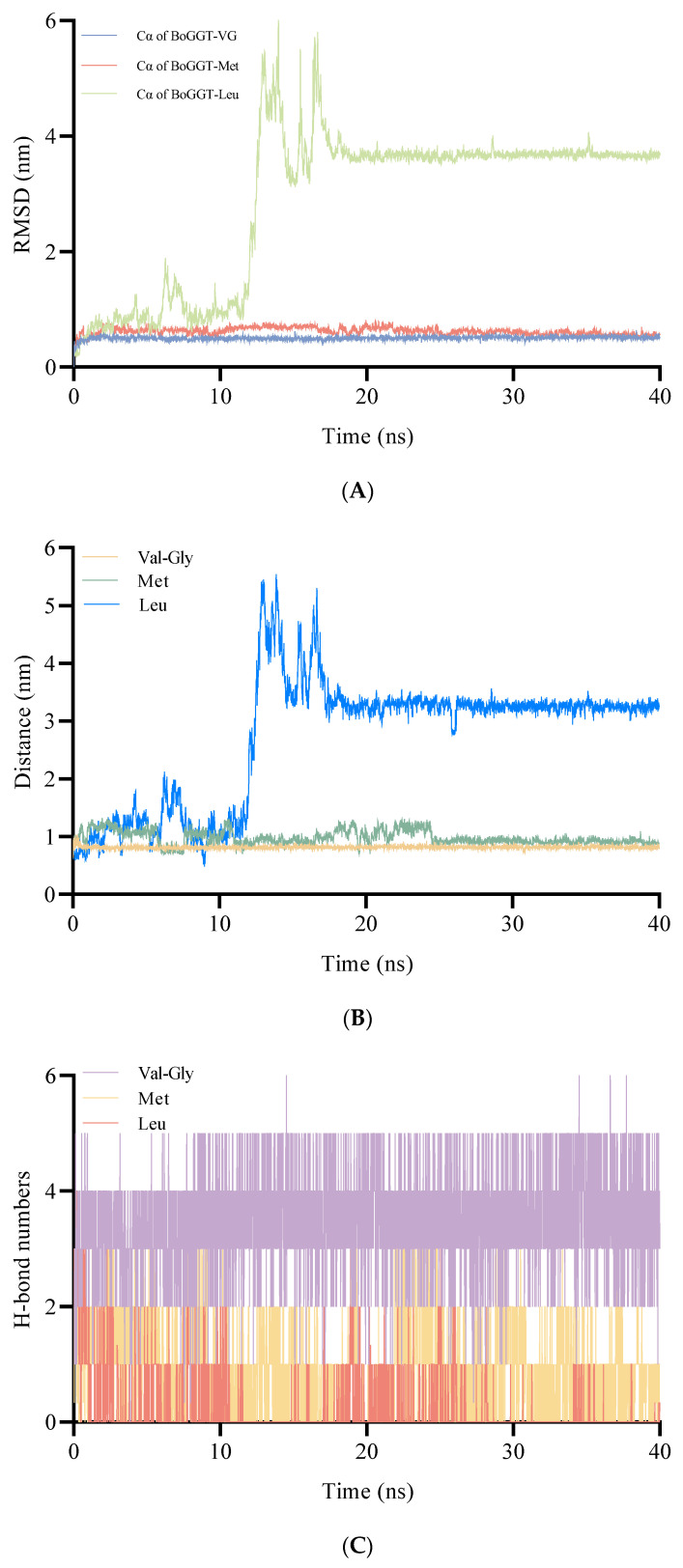
Molecular dynamic simulations of the γ-glutamyl–BoGGT intermediate–acceptor complexes. (**A**) Root-mean-square deviation of Cα of the γ-glutamyl–BoGGT intermediate–acceptor. (**B**) Distance between the hydroxyl oxygen atom on residue380 of the γ-glutamyl–BoGGT intermediate and the nitrogen on the acceptor substrate. (**C**) Hydrogen bond numbers between the γ-glutamyl–BoGGT intermediate and the acceptor substrate.

After the molecular docking, the BoGGT intermediate–acceptor complexes were used to perform 40 ns MD simulations to explore the interaction between the intermediate and acceptors. As shown in Figure 6A, the RMSD of the BoGGT intermediate–acceptor complexes (C_α_ of the BoGGT intermediate–acceptor) stabilized after 20 ns of simulations, indicating that the BoGGT intermediate and the acceptor reached a steady state. 

In the second step, the nitrogen atom on the acceptor will attack the oxygen atom on GGT380 (γ-glutamyl-threonine) to produce γ-glutamyl peptides. Therefore, the distance between the oxygen atom on residue 380 and the nitrogen atom on the acceptor is very important to produce γ-glutamyl peptides. As presented in Figure 6B, during the 30–40 ns simulations, the distance between the oxygen atom (O) on GGT380 and the nitrogen atom on Val-Gly (N) (0.814 ± 0.022 nm) was significantly (*p* < 0.05) lower than that of L-methionine (0.917 ± 0.039 nm) and L-leucine (3.244 ± 0.094 nm), indicating that the intermediate has a higher interaction possibility with Val-Gly. It is generally accepted that H-bonds between enzyme and substrate are the main driving force for the enzymatic reaction. Consequently, the number of H-bonds was also employed to evaluate the interactions between the intermediate and acceptors. As shown in Figure 6C, during the 30–40 ns simulations, the number of H-bonds between the BoGGT intermediate and Val-Gly were significantly higher than that of L-methionine and L-leucine (*p* < 0.05), which could decrease the O-N distance and thus promote the transfer of the γ-glutamyl moiety from the BoGGT intermediate to Val-Gly. To further validate the binding affinity between the acceptor substrate and the BoGGT intermediate, MMPBSA analysis was employed to estimate the binding affinity between the complexes during MD simulations. As shown in Table 1, the total binding energy of the BoGGT intermediate–Val-Gly complexes (−141.062 ± 14.829 kJ/mol) was significantly lower than those of the BoGGT intermediate–Met (−110.784 ± 11.275 kJ/mol) and BoGGT intermediate–Leu complexes (−75.481 ± 19.973 kJ/mol), indicating that BoGGT has a higher affinity for the Val-Gly acceptor. Moreover, the total binding energy of the BoGGT intermediate–Met complexes was significantly lower than that of the BoGGT intermediate–Leu complexes, denoting that BoGGT has a lower affinity for the L-leucine acceptor.

## 3. Materials and Methods

### 3.1. Structure Construction of BoGGT and Molecular Docking between γ-glutamyl Donor and BoGGT

In the current study, the three-dimensional (3D) structure of bovine GGT (UniProt no. G3N2D8) was produced by AlphaFold (v2.0) on an in-house system equipped with an A40 GPU running Docker [19,27]. Multiple sequence alignment was performed against the reduced database (db_preset = reduced_dbs), max_template_date set to the enzymes and model_preset as monomer. Each of the five relaxed structures was manually inspected for defects, and the structure with the highest score was selected for further analysis. 

The 2D structures of the donor substrates (L-glutamine or L-γ-glutamyl-*p*-nitroanilide) were obtained from PubChem, and the 3D structures were converted using ChemSketch (ACD Labs, Toronto, ON, Canada). This allowed us to generate models for donor substrate–enzyme complexes by molecular docking using Autodock Vina with default parameters [28,29]. The docking results of ligand–enzyme complexes were ranked based on the binding energy, and the best match was chosen to analyze the interactions between BoGGT and the donors in PyMOL (Schrödinger Inc., New York, NY, USA).

### 3.2. MD Simulation of BoGGT–Donor Complex

GROMACS (version 2022.2) was utilized to conduct MD simulations for BoGGT–Gln and BoGGT–G*p*NA complexes, based on our previous study [30]. Force field parameters of BoGGT were written in Charmm36 all-atom force field format (July 2021), and force field parameters of L-glutamine and γ-G*p*NA were generated by the CHARMM General Force Field (CGenFF, https://cgenff.umaryland.edu, accessed on 6 September 2022). In the first stage, a steep descent approach of 50,000 steps was adopted to minimize energy and achieve a stable conformation. After minimizing the system’s energy, we performed an isochoric–isothermal equilibration (300 K) for 100 ps, followed by an isothermal–isobaric equilibration (300 K, 1.0 bar) for 100 ps. The complexes were subsequently subjected to 80 ns MD simulations, meaning that the simulations were run in blocks of 80 ns until RMSD simulations were stable within this time course.

The RMSD, RMSF, and H-bond number between BoGGT and donors (L-glutamine and γ-G*p*NA) were calculated after the MD simulations. To evaluate their interactions, the distance between the amide carbon (C) on the donor and hydroxyl oxygen (OG_1_) on the active site was also calculated.

### 3.3. Construction of the BoGGT Intermediate and Optimization of the Intermediate

GGT first interacts with a γ-glutamyl group to form γ-glutamyl–GGT intermediate, which then combines with acceptors to synthesize γ-glutamyl peptides [15]. To reveal the mechanism between the γ-glutamyl–BoGGT intermediate and acceptors, the γ-glutamyl–BoGGT intermediate was constructed and optimized using GROMACS. The γ-glutamyl–BoGGT intermediate was formed when hydroxyl oxygen on the active site of BoGGT (Thr380) attacked the carbonyl carbon of the γ-glutamyl group from the donor. In other words, the γ-glutamyl–BoGGT intermediate is the combination of the hydroxyl oxygen of Thr380 on BoGGT with the γ-glutamyl group from the donor. The γ-glutamyl-threonine is not a standard residue in GROMACS; we need to define it in the existing force field for GROMACS to recognize this residue as an amino acid. Briefly, the atoms and linkage information for the new residue was added to the RTP file (aminoacids.RTP), and then the information about how to add hydrogens to the new residue was supplemented to the HDB file (aminoacids.HDB). Afterward, the information about modifying the residue if it is a terminal residue was added to the r2b file. The new residue was supplemented to residuetypes.dat with the name of GGT into the working directory. The γ-glutamyl-threonine residue was built and aligned with the Thr380 of BoGGT, and then the original Thr380 was deleted to obtain the γ-glutamyl–BoGGT intermediate. Subsequently, a 100 ns MD simulation (300 K, 1.0 bar) was applied to optimize the γ-glutamyl–BoGGT intermediate.

### 3.4. Molecular Docking between the BoGGT Intermediate and γ-glutamyl Acceptors

Molecular docking between the BoGGT intermediate and acceptors was performed using Autodock Vina [28,29]. Briefly, the 2D structures of acceptor substrates (Val-Gly, L-methionine, and L-leucine) were obtained from PubChem, and the 3D structures were converted using ChemSketch (ACD Labs, Toronto, ON, Canada). This allowed us to generate models for acceptor substrate–enzyme complexes by molecular docking using Autodock Vina with default parameters [28,29]. The docking results of ligand–enzyme complexes were ranked based on the binding energy, and the best match was chosen to analyze the interactions between the BoGGT intermediate and acceptors in PyMOL (Schrödinger Inc., New York, NY, USA).

### 3.5. MD Simulation of BoGGT Intermediate–Acceptor Complexes

GROMACS was utilized to conduct MD simulations for BoGGT intermediate–acceptor complexes, based on our previous study [30]. Force field parameters of the BoGGT intermediate were written in Charmm36 all-atom force field format (July 2021), and force field parameters of acceptor substrates were generated by the CHARMM General Force Field (CGenFF, https://cgenff.umaryland.edu, accessed on 15 November 2022). In the first stage, a steep descent approach of 50,000 steps was adopted to minimize energy and achieve a stable conformation. After minimizing the system’s energy, we performed an isochoric–isothermal equilibration (300 K) for 100 ps, followed by an isothermal–isobaric equilibration (300 K, 1.0 bar) for 100 ps. The complexes were subsequently subjected to MD simulations that were run in 40 ns blocks until a time course of relatively stable RMSD was observed. 

RMSDs and H-bond numbers between the intermediate and acceptors were calculated after the MD simulations. To evaluate their interactions, the distance between the amide nitrogen (N) on the acceptor and hydroxyl oxygen on the active site was also calculated. The molecular mechanics Poisson Boltzmann surface area (MMPBSA) was applied to determine the binding free energy using the g_mmpbsa package [31]. Binding energy was calculated using the final 10 ns from the 40 ns MD simulations. Bonded and non-bonded interactions in the vacuum were utilized to estimate the binding affinity of the BoGGT intermediate with acceptors in the solvent stage. The Poisson Boltzmann equation and Solvent Accessible Surface Area (SASA) were utilized to determine the polar and non-polar solvation energy.

### 3.6. Statistical Analysis

The MD simulations were performed in triplicate. The obtained data are presented as mean ± standard deviation. To test differences among means of multiple groups, one-way analysis of variance (ANOVAs) with Duncan’s analysis was utilized. To determine whether the difference in means between the two groups was statistically significant, a *t*-test was used. A *p*-value less than 0.05 (*p* < 0.05) indicates that there are significant differences between the samples. The figures and ANOVAs were created using GraphPad Prism 9.3.1 and SAS 9.4, respectively.

## 4. Conclusions

BoGGT is an enzyme that could produce γ-glutamyl peptides with L-glutamine as a donor substrate and has the potential as a catalyst to produce taste enhancers, whereas the transpeptidase activity is highly dependent on the γ-glutamyl donors and acceptors. In summary, to explore the molecular mechanism behind the donor and acceptor substrate preferences for BoGGT, structure prediction of BoGGT, molecular docking, and molecular dynamic simulations were performed. The light subunit of BoGGT (Thr380, Thr398, Ser450, Ser451, Met452, and Gly473) is responsible for forming the majority of the H-bonds with the L-glutamine donor. Ser450 is a crucial residue for both L-glutamine–BoGGT and G*p*NA–BoGGT interactions. H-bond numbers between L-glutamine and BoGGT were significantly higher than that of γ-G*p*NA, which can decrease the distance between the hydroxyl oxygen atom on the active site of BoGGT and the amide carbon on the donor substrate (OG_1_-C), thereby promoting the binding affinity between BoGGT and L-glutamine. Gly379, Ile399, and Asn400 are crucial residues for the interactions between the BoGGT intermediate and acceptors. H-bond numbers between the BoGGT intermediate and Val-Gly were significantly higher than that of L-methionine and L-leucine, which can decrease the O_1_-N distance, thereby promoting the transfer of the γ-glutamyl group from the intermediate to Val-Gly. This study reveals the interactions between BoGGT and the donor, as well as the interactions between the intermediate and the acceptor substrate, and provides a meaningful method for selecting donors and acceptors with higher affinity for GGT, which can extend the understanding of the catalytic mechanism and promote the yields of γ-glutamyl peptides.

## Figures and Tables

**Table 1:** Total binding energies for acceptor substrates against the BoGGT intermediate.

Complex	Total Binding Energy (kJ/mol)	Van der Waals Energy (kJ/mol)	Electrostatic Energy (kJ/mol)	Polar Solvation Energy (kJ/mol)	SASA Energy (kJ/mol)
BoGGT intermediate–Val-Gly	−141.062 ± 14.829	−176.361 ± 25.742	−102.037 ± 36.825	165.193 ± 41.980	−27.857 ± 5.184
BoGGT intermediate–Met	−110.784 ± 11.275	−157.906 ± 32.609	−76.074 ± 23.259	134.209 ± 33.527	−11.013 ± 4.513
BoGGT intermediate–Leu	−75.481 ± 19.973	−123.524 ± 27.128	−62.598 ± 19.937	124.372 ± 32.252	−13.731 ± 4.235

## Data Availability

Not applicable.

## References

[1] Cao L., Li Q., Lametsch R. (2021). Identification and Activity Characterization of gamma-Glutamyltransferase from Bovine Milk. J. Agric. Food Chem..

[2] Cao L., Li Q., Lametsch R. (2023). Comparative analysis of substrate affinity and catalytic efficiency of gamma-glutamyltransferase from bovine milk and *Bacillus amyloliquefaciens*. Food Chem..

[3] Hillmann H., Behr J., Ehrmann M.A., Vogel R.F., Hofmann T. (2016). Formation of Kokumi-Enhancing gamma-Glutamyl Dipeptides in Parmesan Cheese by Means of gamma-Glutamyltransferase Activity and Stable Isotope Double-Labeling Studies. J. Agric. Food Chem..

[4] Yang J., Bai W., Zeng X., Cui C. (2019). Gamma glutamyl peptides: The food source, enzymatic synthesis, kokumi-active and the potential functional properties—A review. Trends Food Sci. Technol..

[5] Zhao C.J., Schieber A., Ganzle M.G. (2016). Formation of taste-active amino acids, amino acid derivatives and peptides in food fermentations—A review. Food Res. Int..

[6] Lu Y., Wang J., Soladoye O.P., Aluko R.E., Fu Y., Zhang Y. (2021). Preparation, receptors, bioactivity and bioavailability of γ-glutamyl peptides: A comprehensive review. Trends Food Sci. Technol..

[7] Toelstede S., Hofmann T. (2009). Kokumi-active glutamyl peptides in cheeses and their biogeneration by *Penicillium roquefortii*. J. Agric. Food Chem..

[8] Toelstede S., Dunkel A., Hofmann T. (2009). A series of kokumi peptides impart the long-lasting mouthfulness of matured Gouda cheese. J. Agric. Food Chem..

[9] Yang Z., Wang J., Han Z., Blank I., Meng F., Wang B., Cao Y., Tian H., Chen C. (2022). Isolation, identification and sensory evaluation of kokumi peptides from by-products of enzyme-modified butter. J. Sci. Food Agric..

[10] Somma V., Calvio C., Rabuffetti M., Rama E., Speranza G., Morelli C.F. (2021). An overall framework for the *E. coli* gamma-glutamyltransferase-catalyzed transpeptidation reactions. Bioorg. Chem..

[11] Shuai Y., Zhang T., Jiang B., Mu W. (2010). Development of efficient enzymatic production of theanine by gamma-glutamyltranspeptidase from a newly isolated strain of *Bacillus subtilis*, SK11.004. J. Sci. Food Agric..

[12] Blel M., Guingamp M.-F., Gaillard J.-L., Humbert G. (2002). Studies on the thermal sensitivity of γ-glutamyl transpeptidase measured with a modified test procedure and compared with that of alkaline phosphatase and lactoperoxidase in milk. Le Lait.

[13] Baumrucker C.R. (1979). gamma-Glutamyl transpeptidase of bovine milk membranes: Distribution and characterization. J. Dairy Sci..

[14] Tate S.S., Meister A. (1974). Interaction of gamma-glutamyl transpeptidase with amino acids, dipeptides, and derivatives and analogs of glutathione. J. Biol. Chem..

[15] West M.B., Chen Y., Wickham S., Heroux A., Cahill K., Hanigan M.H., Mooers B.H. (2013). Novel insights into eukaryotic gamma-glutamyltranspeptidase 1 from the crystal structure of the glutamate-bound human enzyme. J. Biol. Chem..

[16] Terzyan S.S., Burgett A.W., Heroux A., Smith C.A., Mooers B.H., Hanigan M.H. (2015). Human gamma-Glutamyl Transpeptidase 1: Structures of the free enzyme, inhibitor-bound tetrahedral transition states, and glutamate-bound enzyme reveal novel movement within the active site during catalysis. J. Biol. Chem..

[17] Taylor R.D., Jewsbury P.J., Essex J.W. (2002). A review of protein-small molecule docking methods. J. Comput. Aided Mol. Des..

[18] Sledz P., Caflisch A. (2018). Protein structure-based drug design: From docking to molecular dynamics. Curr. Opin. Struct. Biol..

[19] Jumper J., Evans R., Pritzel A., Green T., Figurnov M., Ronneberger O., Tunyasuvunakool K., Bates R., Zidek A., Potapenko A. (2021). Highly accurate protein structure prediction with AlphaFold. Nature.

[20] Castonguay R., Halim D., Morin M., Furtos A., Lherbet C., Bonneil E., Thibault P., Keillor J.W. (2007). Kinetic characterization and identification of the acylation and glycosylation sites of recombinant human gamma-glutamyltranspeptidase. Biochemistry.

[21] Ikeda Y., Fujii J., Anderson M.E., Taniguchi N., Meister A. (1995). Involvement of Ser-451 and Ser-452 in the catalysis of human gamma-glutamyl transpeptidase. J. Biol. Chem..

[22] Chen D., Oezguen N., Urvil P., Ferguson C., Dann S.M., Savidge T.C. (2016). Regulation of protein-ligand binding affinity by hydrogen bond pairing. Sci. Adv..

[23] Hibi T., Nii H., Nakatsu T., Kimura A., Kato H., Hiratake J., Oda J. (2004). Crystal structure of gamma-glutamylcysteine synthetase: Insights into the mechanism of catalysis by a key enzyme for glutathione homeostasis. Proc. Natl. Acad. Sci. USA.

[24] Okada T., Suzuki H., Wada K., Kumagai H., Fukuyama K. (2006). Crystal structures of gamma-glutamyltranspeptidase from *Escherichia coli*, a key enzyme in glutathione metabolism, and its reaction intermediate. Proc. Natl. Acad. Sci. USA.

[25] Morrow A.L., Williams K., Sand A., Boanca G., Barycki J.J. (2007). Characterization of *Helicobacter pylori* gamma-glutamyltranspeptidase reveals the molecular basis for substrate specificity and a critical role for the tyrosine 433-containing loop in catalysis. Biochemistry.

[26] Wada K., Irie M., Suzuki H., Fukuyama K. (2010). Crystal structure of the halotolerant gamma-glutamyltranspeptidase from *Bacillus subtilis* in complex with glutamate reveals a unique architecture of the solvent-exposed catalytic pocket. FEBS J..

[27] Varadi M., Anyango S., Deshpande M., Nair S., Natassia C., Yordanova G., Yuan D., Stroe O., Wood G., Laydon A. (2022). AlphaFold Protein Structure Database: Massively expanding the structural coverage of protein-sequence space with high-accuracy models. Nucleic Acids Res..

[28] Trott O., Olson A.J. (2010). AutoDock Vina: Improving the speed and accuracy of docking with a new scoring function, efficient optimization, and multithreading. J. Comput. Chem..

[29] Eberhardt J., Santos-Martins D., Tillack A.F., Forli S. (2021). AutoDock Vina 1.2.0: New Docking Methods, Expanded Force Field, and Python Bindings. J. Chem. Inf. Model..

[30] Cao L., Hunt C.J., Lin S., Meyer A.S., Li Q., Lametsch R. (2023). Elucidation of the Molecular Mechanism of Bovine Milk gamma-Glutamyltransferase Catalyzed Formation of gamma-Glutamyl-Valyl-Glycine. J. Agric. Food Chem..

[31] Abdulrashid N.I., Aminu S., Adamu R.M., Tajuddeen N., Isah M.B., Jatau I.D., Aliyu A.B., Simelane M.B.C., Onyike E., Ibrahim M.A. (2022). Phloroglucinol as a Potential Candidate against *Trypanosoma congolense* Infection: Insights from In Vivo, In Vitro, Molecular Docking and Molecular Dynamic Simulation Analyses. Molecules.

